# Telomere Length and the Cancer–Atherosclerosis Trade-Off

**DOI:** 10.1371/journal.pgen.1006144

**Published:** 2016-07-07

**Authors:** Rivka C. Stone, Kent Horvath, Jeremy D. Kark, Ezra Susser, Sarah A. Tishkoff, Abraham Aviv

**Affiliations:** 1 The Center of Human Development and Aging, New Jersey Medical School, Rutgers, Newark, New Jersey, United States of America; 2 Epidemiology Unit, Hebrew University–Hadassah School of Public Health and Community Medicine, Jerusalem, Israel; 3 Department of Epidemiology, Mailman School of Public Health, Columbia University, New York, New York, United States of America; 4 New York State Psychiatric Institute, New York, New York, United States of America; 5 Department of Genetics, School of Arts and Sciences, University of Pennsylvania, Philadelphia, Pennsylvania, United States of America; 6 Department of Biology, School of Arts and Sciences, University of Pennsylvania, Philadelphia, Pennsylvania, United States of America; University College London, UNITED KINGDOM

## Abstract

Modern humans, the longest-living terrestrial mammals, display short telomeres and repressed telomerase activity in somatic tissues compared with most short-living small mammals. The dual trait of short telomeres and repressed telomerase might render humans relatively resistant to cancer compared with short-living small mammals. However, the trade-off for cancer resistance is ostensibly increased age-related degenerative diseases, principally in the form of atherosclerosis. In this communication, we discuss (a) the genetics of human telomere length, a highly heritable complex trait that is influenced by genetic ancestry, sex, and paternal age at conception, (b) how cancer might have played a role in the evolution of telomere biology across mammals, (c) evidence that in modern humans telomere length is a determinant (rather than only a biomarker) of cancer and atherosclerosis, and (d) the potential influence of relatively recent evolutionary forces in fashioning the variation in telomere length across and within populations, and their likely lasting impact on major diseases in humans. Finally, we propose venues for future research on human telomere genetics in the context of its potential role in shaping the modern human lifespan.

## Introduction

In mammals, telomeres consist of TTAGGG tandem repeats and their binding proteins, which together cap and protect the ends of chromosomes [1]. Telomeres experience progressive shortening with successive cell replications, ultimately reaching a critically short length, triggering replicative senescence, which might be one of the mechanisms that drive aging of tissues and organs in vivo [2,3]. A body of data exists on telomere length (TL) dynamics (TL and its age-dependent attrition) and the association of TL with human diseases. These include a number of aging-related disorders that belong to the two disease categories that largely shape the lifespan of humans in high- and middle-income countries: cancer and atherosclerotic cardiovascular disease (CVD). Still, a clear conceptual framework for the role of telomere biology in the evolution of human health and disease is lacking. Moreover, earlier research promoted the view that short TL, as expressed in leukocytes, is a non-causal biomarker of increased risk for aging-related diseases. In contrast, more recent research based on the analysis of genetic determinants of TL and on Mendelian randomization studies using single nucleotide polymorphisms (SNPs) as proxies for TL suggests that TL might play a causal role in a number of cancers [4–6] and in atherosclerotic CVD [7].

## General Characteristics of Telomere Length in Humans

*Homo sapiens* displays short TL compared with most other mammalian species [8] and repressed activity in somatic tissues of telomerase [8–10], the reverse transcriptase that uses its RNA template to add telomere repeats to the DNA termini [1]. During fetal development, a similar TL is maintained across somatic cells [11,12], including cells in the hematopoietic system [13]. That is, fetuses and newborns with short (or long) TL in one tissue display short (or long) TL in other tissues. During postnatal growth and development, some tissues exhibit more TL shortening than others; for example, there is more TL attrition in the highly proliferative hematopoietic system than in much less proliferative muscle tissue. However, the similarity in TL across tissues that has been established by adulthood is largely maintained over the remaining life course [14].

Knowledge of TL dynamics in humans is primarily based on research using leukocytes. Leukocyte TL (LTL) is highly familial with about 65% heritability; the heritability of age-dependent LTL attrition (in adults) is about 30% [15–18]. In addition, sex (females have longer LTL than males [16,19,20]), ancestry (African Americans [21] and sub-Saharan Africans [22] have longer LTL than individuals of European ancestry), and father’s age at conception (offspring of older fathers have longer LTL than those of younger fathers [23–26]) also influence LTL. Environmental factors, including socioeconomic status [27], smoking [19,28], sedentary lifestyle [29], energy intake [30], and perhaps mental stress [31,32] might be associated with a short LTL, suggesting that these factors might influence or be influenced by LTL dynamics to some extent.

On average, mean LTL at birth is 9.5 kb in Europeans with a wide range of variation across newborns [11,33,34]. Although LTL undergoes progressive shortening with age, the LTL variance observed in newborns is largely maintained during the life course [11,14]. At the age of 20 years, mean LTL is about 7.8 kb. Thereafter, the average LTL attrition approximates 25 base pairs per year [35]. Little is known about inter-individual variation in LTL attrition during growth and development based on longitudinal study design. Longitudinal studies show that LTL attrition during adulthood does vary across individuals within a given population, but for the overwhelming majority of the population, the inter-individual variation in this attrition is typically of a magnitude that is insufficient to produce a substantial change in an individual’s LTL percentile ranking compared with peers [36]. Based on the findings in fetuses [12], newborns [11,13,33,34], and adults [14,36,37], and on modeling of LTL attrition during growth [38], we infer that LTL at birth is a major determinant of LTL in adults, such that newborns with short (or long) LTL are likely to have short (or long) LTL throughout their life course [39,40].

## The Genetics of Leukocyte Telomere Length

LTL is a complex genetic trait. A series of population-based genome-wide association studies (GWAS) has identified genetic loci associated with LTL. To date, 11 LTL-associated loci have been identified in cohorts of Europeans [7,41–44], and many more genetic loci associated with LTL remain to be identified and characterized across populations. Some of the loci that have already been found to be associated with LTL harbor genes that are engaged in telomere maintenance. These include genes encoding the two major subunits of telomerase, the telomerase reverse transcriptase (*TERT*) and its RNA component (*TERC*), two of the members of the human CST (CTC1, STN1, and TEN1) complex, i.e., *CTC1* and *STN1* (*OBFC1*), telomerase ribonucleoprotein nuclear assembly factor 1 (*NAF1*), and telomere length maintenance gene regulator of telomere helicase 1 (*RTEL1*) [7,41,42,44]. The exact roles of several other LTL-associated genes in TL regulation are poorly understood; these include zinc finger proteins *ZNF208* and *ZNF676*, neutrophil trafficking chemokine *CXCR4*, and *DCAF4*, which is involved in the ultraviolet radiation-induced DNA damage response [7,42–44].

Major germ line mutations can cause critically short telomeres that might lead to bone marrow failure. For example, mutations in *TERT* and *TERC* as well as in telomere end-protective shelterins (telomeric repeat binding factor 1 [*TERF1*] and TRF1-interacting nuclear factor 2 [*TINF2*]) lead to extremely short telomeres and the development of dyskeratosis congenita and idiopathic pulmonary fibrosis, which typically present with aplastic anemia [45]. However, these telomeropathies are uncommon and may not explain telomere-related diseases in the general population.

## Cancer as an Evolutionary Force Affecting Telomere Length

TL genetics should be considered in the context of evolutionary forces that have left their signature on the human genome. Inspection of the human genome reveals that of the approximately 22,000 currently annotated genes, 13,000 genes (about 60%) are linked to biological pathways of “cancer” (as referenced in the Ingenuity Knowledge Base [www.ingenuity.com]). These include genes engaged in growth, development, tissue regeneration, and tissue renewal, which heighten cancer risk due to increased cell replication, and genes that suppress cancer, including those that ultimately promote senescence and apoptosis [46]. Central among cancer-protective pathways might be telomere-driven replicative senescence [47].

Stem cells are likely to undergo more replications in large, long-living mammals than in small, short-lived ones; this is because more replications are necessary for developing and maintaining a larger body size. More cell replication confers increased risk of cancer through accumulating de novo somatic mutations, which happen during successive DNA replications. This concept is supported by work showing that the risk of developing major human cancers is related to the number of stem cell divisions occurring in the tissues from which the cancers originated [48,49]. Yet, large, long-living mammals generally display no increase in cancer risk compared to small, short-lived ones, a phenomenon known as Peto’s paradox [50], suggesting that mechanisms have evolved to mitigate cancer risk in tandem with increasing body size and longevity [51,52]. One such mechanism has been described in elephants. The elephant genome contains many more copies of *TP53*, a potent DNA damage response and tumor suppressor gene; p53-dependent apoptosis is thus triggered at a lower threshold of accumulating mutations, conferring cellular resistance to oncogenic transformation [53].

Similarly, a telomere-linked mechanism has been proposed in mammals based upon observations that telomerase activity in somatic tissues tends to be inversely correlated with body size [9,10] while TL is inversely related to lifespan [8]. Repressed telomerase and short telomeres would, therefore, limit replicative capacity in humans. In this way, short TL might curb the accumulation of de novo mutations and reduce the probability of oncogenic transformation in large, long-living mammals.

Given the wide variation in TL across humans, might longer telomeres increase cancer risk? While initial studies were inconsistent (perhaps partially due to flaws in study design and small sample sizes [54]), more recent studies show that in individuals of European ancestry, long telomeres, as expressed in LTL, are associated with increased risk for melanoma [4,55,56], adenocarcinoma of the lung [57–59], and cancers of the breast ([60], but see [61]), pancreas [62], and prostate [63]. Moreover, Mendelian randomization studies using LTL-associated SNPs support the inference that having a longer LTL has a causal relation to cancer risk [5,6].

## Polygenetic Adaptation and Telomere Length

Complex traits that are influenced by multiple genetic loci may be targets of polygenic adaptation [64,65], i.e., simultaneous shifts in frequencies of many alleles influencing a trait in response to local adaptation during human evolutionary history. Collectively, however, these alleles might ultimately explain a large proportion of the wide variation in LTL across humans. An illustration of this concept comes from findings that individuals of European ancestry display shorter LTL than sub-Saharan Africans and African Americans, and that loci associated with LTL show evidence of polygenic adaptation in Europeans compared to Africans [22].

Dark skin renders protection to Africans from the DNA-damaging effect of ultraviolet radiation (UVR); such damage is a major risk factor for melanoma [66]. The northbound migration out of Africa approximately 20–40 thousand years ago was ostensibly associated with skin depigmentation and thus increased sensitivity to UVR, which might have augmented the risk of melanoma. The idea that melanoma acted as an evolutionary selective force in ancestral humans has been challenged based on the assumption that the life span of ancestral humans was relatively short, and melanoma principally strikes individuals during the post-reproductive years [67]. However, this view does not consider that once surviving childhood, ancestral humans lived much longer than originally thought, and considerable evidence indicates that melanoma afflicts adults throughout the life course, including young adulthood [68]. One mechanism whereby individuals with light skin might have attenuated the risk of melanoma is by shortening their telomeres through polygenic adaptation, thereby explaining in part the shorter LTL in individuals of European ancestry than in sub-Saharan Africans [22]. Simply put, Europeans would have been even more susceptible to melanoma without polygenetic adaptation-mediated TL shortening.

## The Cancer–Atherosclerosis Trade-Off

The cancer protection conferred by short telomeres could come with an evolutionary trade-off [69], namely, diminished proliferative activity of stem cells and consequently less regenerative capacity. This would manifest in age-dependent degenerative diseases. Some of the leading degenerative diseases in humans are related to atherosclerosis, and atherosclerosis is associated with short LTL [40,70,71]. As cancer and atherosclerosis strongly impact longevity, the diametrically opposing roles of TL in these two disorders might be relevant to understanding the lifespan of contemporary humans and future trajectories in life expectancy. Notably, in evolutionary terms, this would probably have become more relevant when agrarian societies emerged over the past ten thousand years or so and lifespans increased considerably. In fact, evidence of atherosclerosis has been detected in ancient human Egyptian mummies [72].

**Fig 1:**
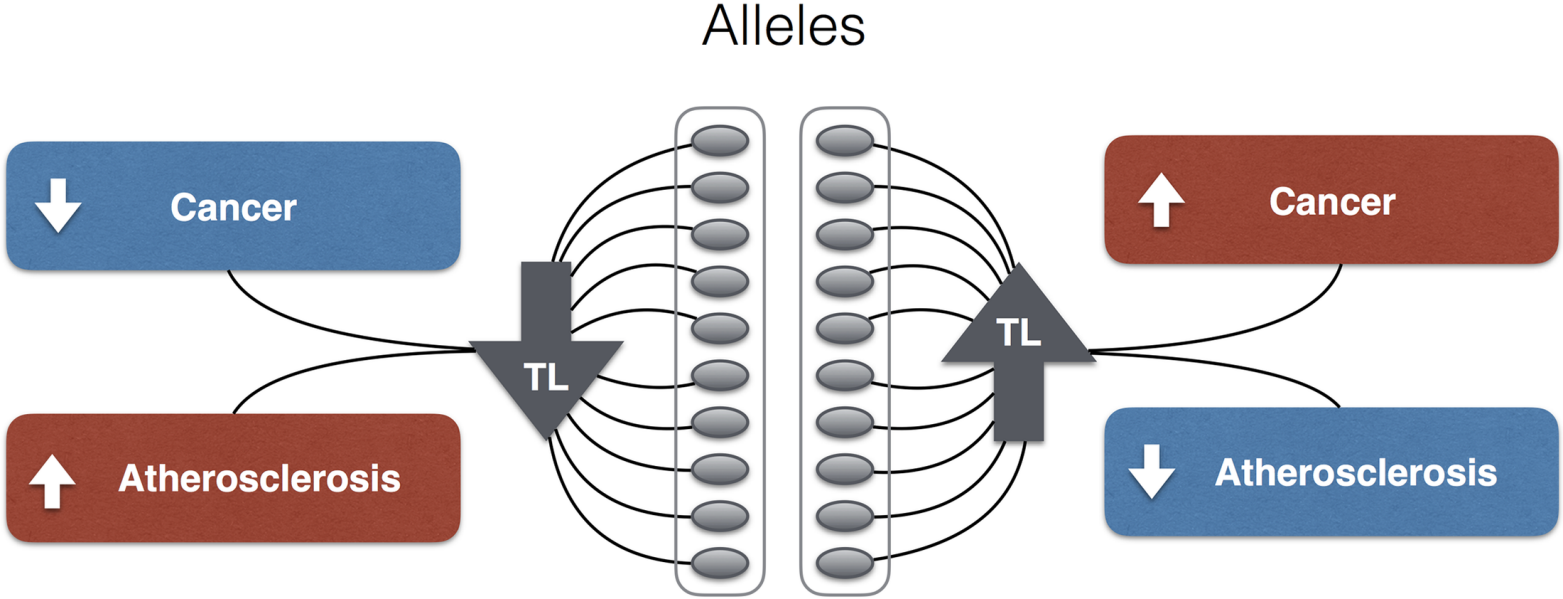
Telomere length (TL) and the cancer–atherosclerosis trade-off at the genomic level. Alleles that are jointly associated with relatively short TL diminish the risk for major cancers and increase the risk for atherosclerosis, while alleles (of the same SNPs) that are jointly associated with relatively long TL increase the risk for major cancers and diminish the risk for atherosclerosis.

Recent studies have used LTL GWAS findings to generate “genetic risk scores” for cancers and for atherosclerosis insofar as it is expressed in coronary heart disease. These studies have shown that the same cluster of LTL-associated alleles (*OBFC1*, *ZNF208*, *TERC*, *TERT*, *RTEL1*, *ACTP2*, and *NAF1*) is a risk indicator for melanoma [4], lung cancer [57], and coronary heart disease [7], such that when the joint effect of the alleles results in a comparatively long LTL, the risk for melanoma and lung cancer is increased, whereas the risk for coronary heart disease is diminished. The opposite holds when the joint effect of the alleles results in a comparatively short LTL, which engenders a higher risk for coronary heart disease and a lower risk for cancer (Fig 1). This cancer–atherosclerosis trade-off might principally apply to contemporary humans because they live so long, but not to ancestral humans.

## Ancestry, Telomere Length, and the Cancer–Atherosclerosis Trade-Off

In light of the longer LTL in African Americans than in individuals of European ancestry, it is of interest to compare the incidence of major cancers and atherosclerosis in the two ethnicities. The incidence of cancers of the lung (after adjustment for smoking), pancreas, and prostate is higher in African Americans than in individuals of European ancestry [73–75]. The incidence of triple-negative breast cancer is also higher in African American women, and its onset is at a younger age than in women of European ancestry [76]. In contrast, in spite of increased risk factors for atherosclerosis due to higher rates of type 2 diabetes, hypertension, left ventricular hypertrophy, and low socioeconomic status, African Americans display a lower incidence of atherosclerosis, as expressed in coronary artery calcification and coronary artery disease [77,78].

## Conclusions and Suggestions for Future Research

Converging lines of evidence point to a causal role of TL in the cancer–atherosclerosis trade-off across humans. This trade-off has been principally established through the force of evolution. In contrast to cancer, which is associated with over ten thousand genes, only several hundred genes in the human genome have been shown to relate to atherosclerosis. Yet, atherosclerosis is a major determinant in the longevity of humans because of their relatively long lifespan, especially in high- and middle-income societies.

We note, however, the paucity of comparative LTL data by ethnicity and that LTL GWAS have been almost exclusively performed on individuals of European ancestry. Moreover, the role of TL in the cancer–atherosclerosis trade-off has been shown thus far based on a few types of cancers and an aggregate of less than a dozen SNPs. There are likely numerous other SNPs throughout the genome that are associated with TL, and distinctive TL-mediated evolutionary trade-offs have probably emerged within different global populations and at different periods to accommodate their unique environmental settings. Therefore, large-scale measurements of LTL and genome-wide genotyping (sequencing) of different ethnic groups residing in diverse geographical regions across the globe will be essential to decipher the evolutionary signature in human telomere biology and to gain further insight into the role of telomeres in human diseases.
